# Hospitalization and mortality outcomes among adult persons living with HIV in a tertiary hospital in South-western Nigeria: A cross-sectional study

**DOI:** 10.1371/journal.pgph.0003487

**Published:** 2024-07-11

**Authors:** Ayanfe Omololu, Asukwo Onukak, Mfon Effiong, Olaide Oke, Samson E. Isa, Abdulrazaq G. Habib

**Affiliations:** 1 Department of Internal Medicine, Federal Medical Centre, Abeokuta, Nigeria; 2 Department of Internal Medicine, University of Uyo, Uyo, Nigeria; 3 Akwa Ibom State Hospital Management Board, Uyo, Nigeria; 4 Department of Medicine, University of Jos, Jos, Nigeria; 5 Department of Medicine, College of Health Sciences, Bayero University, Kano, Nigeria; Universidad de Chile, CHILE

## Abstract

HIV infection continues to be a major public health issue, with significant morbidity and mortality especially in resource poor areas. Infection with HIV results in an increased risk of opportunistic infections and other complications, which may lead to hospital admission and death. Morbidity and mortality patterns among hospitalized persons living with HIV (PLHIV) have been well documented in high income countries, but there is paucity of such data in Nigeria. We investigated the reasons for hospitalization and predictors of death among adult PLHIV at the Federal Medical Center (FMC) Abeokuta, Nigeria. This was a hospital based cross-sectional study carried out over a 15-month period between January 2018 and March 2019. All consenting hospitalized adult PLHIV who met the inclusion criteria were enrolled into the study. Causes of hospitalization and death were obtained and analyzed. Over the study period, 193 hospitalizations of PLHIV were studied. Although a number of clinical syndromes were documented, Sepsis and Tuberculosis were the commonest causes of hospitalization and mortality. Mortality rate was 37(19.2%) for outcomes on day 30, with anaemia [OR 3.00 (95% C.I: 1.04–8.67)], poor adherence with Cotrimoxazole [OR 4.07 (95% C.I: 1.79–9.28)], poor adherence with cART [OR 13.40 (95% C.I: 3.92–45.44)], and a longer duration of fever [OR 3.34 (95% C.I: 1.10–9.99)] being predictors of mortality. Part of the study’s limitation was resource-constraint of some of the indigent patient which affected their ability to access some diagnostic investigations and get optimal care thereby impacting on their outcome. Despite the upscaling of cART, opportunistic infections and sepsis remain common causes of hospitalization and death in adult PLHIV. More attention should therefore be placed on early diagnosis, prevention of immunosuppression and sepsis through timely administration and adherence to cART and other prophylactic measures.

## Introduction

Human immunodeficiency virus (HIV)-related illnesses were responsible for 690,000 deaths globally, with about three-fifths of these occurring in Sub-Saharan Africa in 2019 [1]. Nigeria has one of the highest burden of HIV/AIDS in the world, with results from the recently concluded Nigeria National HIV/AIDS Indicator and Impact Survey (NAIIS) indicating a prevalence of 1.4% for adults aged 15–49 years, with an estimated population of people living with HIV being 1.9 million [2]. This is a major improvement from the population-based survey conducted by the Nigerian Agency for the control of AIDS (NACA) in 2013, where a HIV prevalence rate of 3.4% was reported [3], with the estimated number of new infections and HIV/AIDS-related deaths being 390,000 and 217,000 respectively. HIV-related conditions make up a large percentage of adult medical admissions in SSA. Studies carried out in Nigeria found that HIV-associated illness was responsible for between 6.7% to 13.1% of medical admissions, and 16.6% to 28% of total deaths [4–6].

Infection with HIV leads to progressive cell-mediated immunodeficiency, resulting in an increased risk of opportunistic infections (OIs) and death. As the CD4+ cell count declines and the viral load increases, PLHIV become increasingly susceptible to OIs that may result in hospital admissions [7–9]. Several factors such as pulmonary tuberculosis, bacterial infections, late presentation to health care facilities, combination antiretroviral therapy (cART)-related complications, and the immune reconstitution inflammatory syndrome (IRIS) have been implicated in causing hospitalization and mortality in PLHIV [10,11]. There has also been an increase in the manifestation of non-communicable diseases (NCDs) such as cardiovascular, pulmonary and hepatic diseases; and non-AIDS malignancies following improved survival of these patients from increased availability of cART [12,13].

As at 2019, about 1.1 million PLHIV out of the estimated 1.9 million were on treatment, giving a 60% cART coverage according to the current Nigerian national HIV prevention, treatment and care guidelines [14]. Poor adherence to cART have been associated with having low CD4+ and attendant predisposition to OIs [15]. In Nigeria, there is a dearth of studies regarding the morbidity and mortality patterns of PLHIV, both in the pre-antiretroviral therapy era and since the massive scale up of cART that began in early 2001 [10,16]. This study seeks to describe the current causes of hospitalization and predictors of mortality among hospitalized adult PLHIV, at a major tertiary health facility in southwest Nigeria since cART became widely available.

## Methods

### Study site

The study was carried out at the Federal Medical Center Abeokuta; a 350-bed referral hospital in Ogun state, Southwestern Nigeria. Over 3000 non-pregnant adults living with HIV are currently under care in the outpatient clinic, from which admissions commonly occur into the medical wards for various reasons.

### Study design and population

This hospital-based cross-sectional study was carried out over a 15-month period between January 3, 2018 and March 30, 2019. All HIV-related admissions into the medical wards during this period were recorded, after obtaining consent, with their routes of admission, presenting complaints, medical diagnosis, duration of hospitalization, and causes of death if mortality occurred. Exclusion criteria included those who declined consent or admission, pregnant women and those admitted for less than a day. Old and new persons living with HIV were sampled if they met the study criteria. Presenting complaints and clinical diagnosis was obtained through detailed medical history, physical examination and appropriate laboratory investigations. Final diagnosis was obtained at discharge, while the cause of death was documented for patients who died during hospitalization. Final outcome on day 30 was determined if the PLHIV was still on admission in the hospital, or via phone follow up if the PLHIV had been discharged.

### Sample size

This was estimated using the Leslie Kisch formula for cross-sectional studies, with a 13% prevalence of HIV-associated admissions (an average from previous studies), and a 0.05 level of precision. Allowance was made for a 10% non-response rate, with a final sample size estimation of 193.

### Study procedure

Patient’s demographic characteristics, clinical presentation and examination findings were recorded. The data were extracted directly from the medical records of the hospitalized patients after obtaining informed consent. Blood samples were collected by venipuncture for full blood count, liver and renal function tests, and CD4+ count measurement, which was done in accordance with the 2016 Nigerian national guidelines for HIV prevention, treatment and care [17]. The 2016 guidelines were the penultimate national guide that was being used during the study duration. The guidelines followed existing evidence-based interventions to improve efficiency in care of PLHIV. The key recommendations in the then guidelines included the introduction of cART in all PLHIV following diagnosis with HIV (test and treat strategy) and the addition of Dolutegravir to the pool of approved antiretroviral medications.

Additional tests to aid in determining the cause of hospitalization was performed at the discretion of the attending clinician, including GeneXpert test, radiological and histological investigations. Tuberculosis was confirmed with a positive GeneXpert assay or sputum acid fast bacilli (AFB) smear in the presence of compatible clinical findings. Diagnosis of HIV was done using the rapid tests first, followed by confirmatory tests (Western blotting), after appropriate confidential counselling. All CD4+ cell counts included in study analyses were either done on current hospitalization, or within the previous 3 months before hospitalization.

Aetiological diagnoses were based on compatible clinical presentation, response to therapy, and/or confirmatory investigations. Sepsis was defined based on clinical features and laboratory investigations, as well as the criteria from the Third International Consensus definitions for sepsis and septic shock (Sepsis-3), using quick Sequential Organ Failure Assessment (qSOFA) score to identify patients requiring further detailed evaluation with a sepsis workup [18]. Patients with new onset focal deficits, were classified as having cerebrovascular disease, if clinical features were in keeping with a vascular cause, and neuro-imaging was suggestive, otherwise, they were classified as a space occupying lesion (SOL) or a stroke like syndrome.

### Definition of survival outcomes

- Survived: If discharged from hospital, discharged against medical advice (DAMA), transferred or alive in hospital on day 30 of admission. (Those discharged before day 30 were followed up via phone contact. The first author oversaw patient follow-up during data collection. Participants data were anonymized after data collection.)
- Died: If dead within 30 days of admission in the hospital.

### Statistical analysis

The Statistical Package for the Social Sciences (SPSS) Version 24 (Armonk, NY: IBM Corp) was used to analyze data. Demographic and baseline clinical and laboratory characteristics was summarized into frequencies, medians and interquartile ranges (IQR). Causes and outcomes of hospitalization was summarized into frequencies and proportions. Descriptive statistics including mean and standard deviations for continuous variables with normal distribution was presented, while the median and range was used to describe continuous but skewed variables. Categorical variables were presented as proportions and statistical comparisons made using the Pearson’s chi–squared test (χ^2^). Baseline clinical and laboratory parameters were cross-tabulated with survival outcomes (dead or survived) to assess for statistical significance. Variables with statistical significance were then subjected to a binary logistic regression analysis, to determine independent predictors of mortality. The results of the binary logistic regression analysis were presented as adjusted odds ratio (aOR) with 95% confidence interval (CI). The predictors of mortality were summarized in a univariate and multivariate analysis. For all statistical comparisons, a probability value (p value) of < 0.05 was considered statistically significant.

### Ethical considerations

Institutional ethics approval was obtained a-priori from Federal Medical Center Abeokuta, Nigeria with approval number FMCA/470/HREC/03/2017. Formal written consent was obtained from study participants.

## Results

### Sociodemographic characteristics of the study participants

About half (100,51.8%) of the PLHIV recruited into the study were between the ages of 41 and 65. The median age of the study participants was 42 years (IQR: 40–44) and there were more females (120, 62.2%). Median body mass index (BMI) was 21 kg/m^2^ and most of the participants (147, 76.1%) earned 30,000 naira monthly income or less. These are summarized in Table 1.

**Table 1:** Sociodemographic characteristics of the study participants.

Demographic variables	Frequency	Percentage (%)
***Age group***		
≤40	90	46.6
41–65	100	51.8
>65	3	1.6
***Median age*** ***Gender***	42(40–44)	
Male Female	73 120	37.8 62.2
***Marital status***		
Married	129	66.8
Single	23	11.9
Widowed	21	10.9
Divorced	20	10.4
***Educational level***		
None	27	14.9
Primary	69	38.1
Secondary	41	22.7
Tertiary	44	24.4
***Occupation***		
Farmer	6	3.1
Artisan	41	21.2
Petty trader	69	35.8
Business	26	13.5
Civil servant	24	12.4
Unemployed	7	3.6
Others	20	10.4
***Monthly income***		
≤N30,000	147	76.1
>N30,000	46	23.9
***Ethnicity***		
Igbo	13	6.7
Hausa	6	3.1
Yoruba	163	84.5
Others	11	5.7
***Religion***		
Christianity	127	65.8
Islam	64	33.2
Traditional	2	1.0

### Causes of hospitalization among study participants

**Fig 1:**
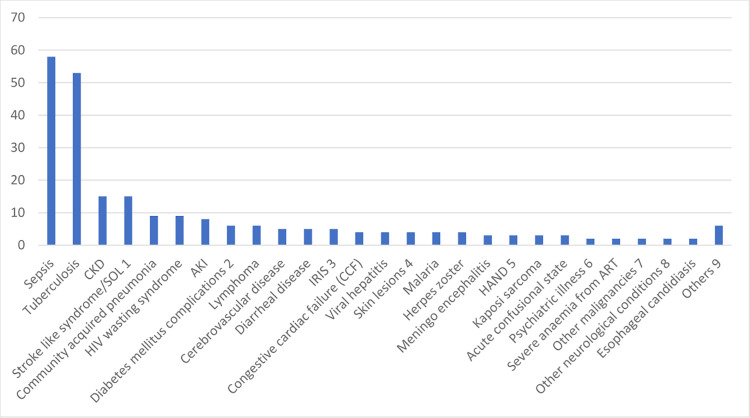
Causes of hospitalization among study participants.

Sepsis (58, 24%) was the most common cause of hospitalization, followed closely by tuberculosis (53, 21.9%). Other causes of hospitalization included stroke like syndromes/space occupying lesions, Chronic kidney disease (CKD), HIV wasting syndrome, community acquired pneumonia, Acute kidney injury (AKI), and lymphoma, among others. Patients were diagnosed with CKD if they had documented functional or structural deterioration in kidney function for at least 3 months, while those with similar deterioration less than 3 months were diagnosed with AKI [19]. These findings are presented in Fig 1.

### Study outcomes of participants

As presented in Table 2, majority (164, 85%) of study participants survived. Average duration of hospitalization was 9 days, while median time to mortality was 5 days. For outcomes on day 30, 37 (19.2%) were dead overall with a mortality incidence rate of 192/1000 hospitalized patients.

**Table 2:** Study outcome of participants.

Outcome	
	Frequency(%)
***Outcome***	
Discharged	147 (76.2)
DAMA*	16 (8.3)
Died	29 (15.0)
Referred	1 (0.5)
***Average duration of hospitalization (days)*** ***Study outcome*** ***(during hospitalization)***	9 (8–11)
Survived	164 (85.0)
Dead	29 (15.0)
***Median time to mortality (days)*** ***30-day outcome***	5 (4–7)
Alive	156 (80.8)
Dead	37 (19.2)
***30-day mortality incidence rate***	192/1000 hospitalized patients

*Discharge against medical advice.

### Causes of death among study participants

**Fig 2:**
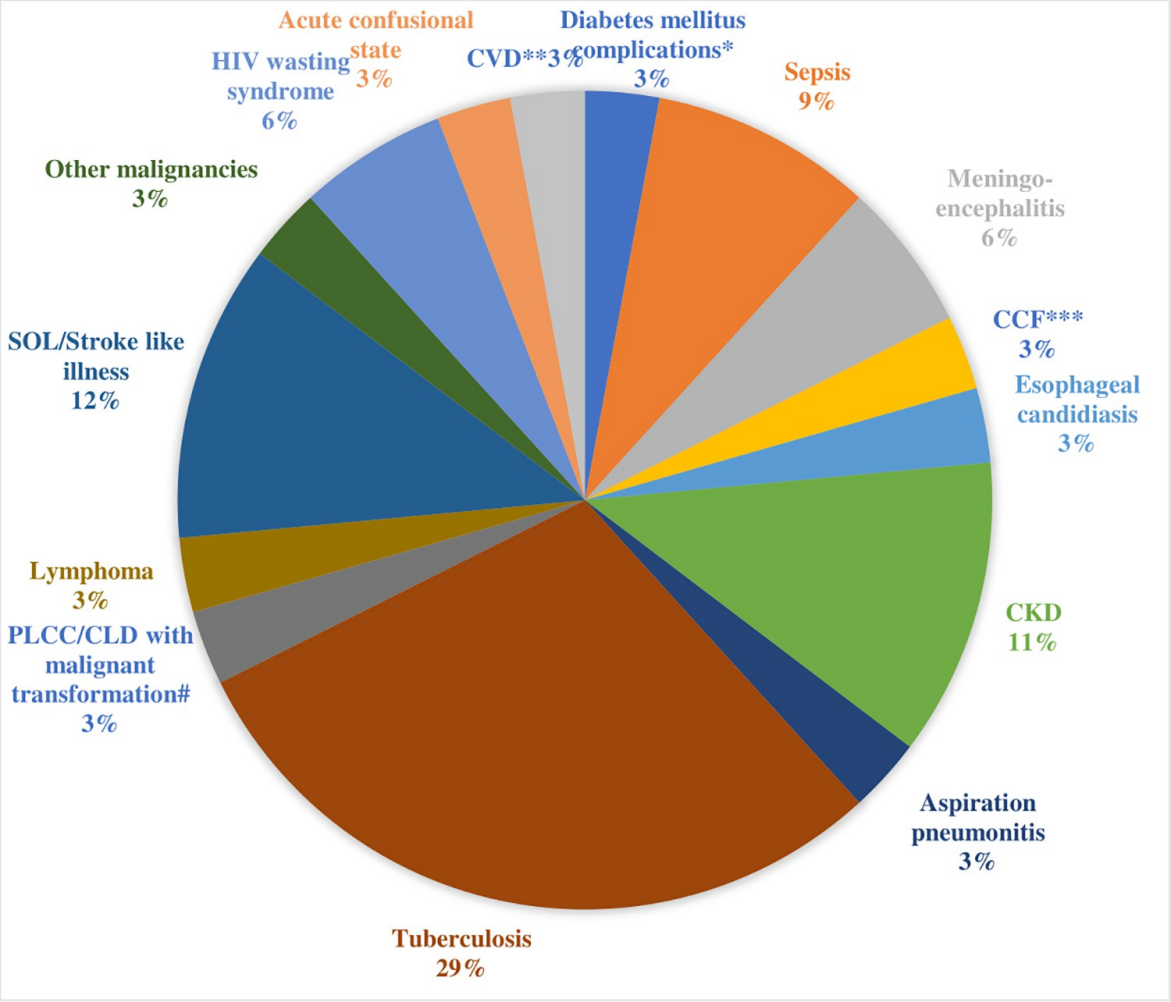
Causes of death among study participants.

Tuberculosis (10, 29%) was the most common cause of death among the PLHIV who participated in the study. Other causes of death included stroke-like syndromes/space occupying lesions, CKD and sepsis among others. These findings are presented in Fig 2.

### Variables associated with mortality in hospitalized PLHIV

The predictors of mortality among hospitalized PLHIV are summarized in Table 3. 107 PLHIV were on cART of which 91 (85%) were adherent. On univariate analysis, anaemia (OR 3.00), non-adherence with cART (OR 13.40), non-adherence with cotrimoxazole (OR 4.07), and a longer duration of fever (OR 3.34), were significantly associated with mortality. However, none of these variables were statistically significant on multivariate regression analysis.

**Table 3:** Variables associated with mortality in hospitalized PLHIV.

Predictors	Univariate analysis		Multivariate analysis	
	OR (95% C.I.)	P-value	AOR (95% C.I.)	P-value
Weight loss	3.10(1.37–6.98)	0.007	0.34(0.05–2.57)	0.298
Patient non-ART adherent	13.40(3.92–45.44)	0.001	3.97(0.42–37.87)	0.231
Non-cotrimoxazole adherent	4.07(1.79–9.28)	0.001	2.26(0.29–17.72)	0.437
PCV (<24%)	3.00(1.04–8.67)	0.042	0.37(0.06–2.26)	0.280
Longer duration of fever	3.34(1.10–9.99)	0.033	0.90(0.12–6.78)	0.920

OR: Odds ratios; AOR: Adjusted odds ratio; CI: Confidence interval. Hosmer and Lemeshow test demonstrated no evidence of lack of fit for significant variables.

## Discussion

Human Immunodeficiency Virus (HIV)-related hospitalization accounted for about 18.8% of total medical admissions in this study, higher than the 5.9% to 13.1% reported from other studies in Nigeria [4–6]. A majority (61%) of the hospitalized PLHIV were young individuals between the ages of 15 to 45 years, in keeping with findings from the 2018 Nigeria HIV/AIDS Indicator and Impact Survey (NAIIS) which showed that about 75% of PLHIV in the country are between the ages of 15 and 49 years [2]. There were also more females among our patients, in keeping with findings from other studies [20] and also from the NAIIS which showed that women aged 15–49 years are more than twice as likely to be living with HIV than men (1.9% versus 0.9%.), while young women aged 20–24 years are more than three times as likely to be living with HIV as young men in the same age group [2], with an expectedly increased morbidity resulting from increased prevalence. Some other studies however, have reported more male patients among hospitalized PLHIV [10].

Although a wide spectrum of clinical manifestations were documented, sepsis and tuberculosis were the most common causes of hospitalization among our patients, similar to findings from other studies [4,10,21], with stroke like syndromes, CKD, HIV wasting syndrome, non-tuberculous pneumonia, lymphoma, IRIS and diarrheal disease making significant contributions. This is in keeping with various studies that have been carried out around the country, across Africa and internationally, showing that despite the upscaling of cART, OIs are still a major cause of morbidity and mortality among HIV/AIDS patients [22–24]. Late diagnosis and a delay in commencement of cART could lead to reduced cell mediated immunity, and a decreased ability of the immune system in PLHIV to fight against common pathogens [25]. It is also important to note that these OIs can be the cause of sepsis. The WHO’s global review of causes of hospitalization among adults living with HIV/AIDS in 2015, showed two disease categories, AIDS-related illnesses and bacterial infections, as the leading causes of hospitalization across all geographical regions [24]. Habib et al in studies carried out in northern Nigeria among PLHIV not on cART, noted that TB was the commonest cause of adult hospitalization, accounting for 29% of HIV admissions in Zaria [7], as PLHIV are much more vulnerable to infection and reactivation. Ogoina et al, also in Zaria, found that although a variety of clinical manifestations were observed, tuberculosis, sepsis, and chronic diarrhoea were the most common cause of hospitalization [10]; findings very similar to this study. A study in Jos, also corroborated these with the findings that pulmonary and extra pulmonary tuberculosis were the most common reasons for hospitalization [20]. Morbidity and mortality risks of TB significantly decrease with cART but remain higher in the first 6 months of initiation than during the subsequent time on therapy [26]. Tuberculosis was the second most frequent cause of morbidity (38%) in West Africa [27], and its incidence remains high in TB-high-burden countries even with cART use. A study in Uganda, also found the most frequent causes of hospitalization were tuberculosis, cryptococcal meningitis, zidovudine (AZT)-associated anaemia, sepsis and Kaposi’s sarcoma. The incidence of extra-pulmonary tuberculosis and PTB were quite similar among our patients, again reflecting the role of immunosuppresion in facilitating the spread of extrapulmonary TB. Tuberculosis was also the most common comorbid condition, similar to other studies [28].

In terms of mortality, PLHIV made up 19.2% of total deaths (by day 30), similar to findings from other local studies, which reported a rate of 16.6–40 [4–6]. Tuberculosis was by far the most common cause of death, in keeping with findings from the earlier listed local and regional studies [7,10,20,28]. Tuberculosis was also the commonest cause of death in hospitalized adult PLHIV in sub-Saharan Africa (Cote d′Ivore, Kano-Nigeria, Zaire) [27,29,30]. Similarly, TB was the third major cause of in-program deaths in South Africa; although in rural Malawi, it was not found to be a risk factor for high early mortality in patients on cART [31,32]. There is significant synergy between HIV and TB such that they both facilitate the progression of each other, especially for patients not on, or poorly adherent on cART. This could be responsible for the high mortality in this cohort. Other common causes of mortality that was found in these group of patients includes Stroke-like syndromes/Space-occupying lesions, CKD, sepsis, HIV wasting syndrome, meningo-encephalitis and lymphoma. Most of the other studies also list sepsis as one of the commonest causes of mortality among HIV patients. Again, poor host immune response in PLHIV could lead to rapid progression of an infection with associated organ dysfunction and death. Sepsis is a medical emergency [18], and needs to be addressed as such. Failure to identify sepsis early, carry out diagnostic investigations and commence appropriate interventions could be responsible for the high incidence of sepsis as a cause of mortality among hospitalized adult PLHIV. Overall, opportunistic infections were the most common diagnostic category, as well as the commonest cause of death in our patients, followed closely by sepsis/bacterial pneumonia, while nearly a fifth of patients presented with AIDS-defining conditions, although these were not mutually exclusive.

It is notable that CKD was the fourth most common cause of hospitalization amongst our patients, reflecting the increasing role that NCDs are playing since the upscaling of cART. Non-communicable diseases have emerged as an important cause of morbidity in both untreated and treated HIV infection. It has been postulated that cART-related complications, HIV-disease itself, or traditional risk factors are responsible for the growing burden of NCDs in people living with HIV [33]. The predictors of mortality were summarized in both univariate and multivariate analysis. On univariate analysis, non-adherence with cART, non-adherence with Cotrimoxazole, anaemia, and a longer duration of fever were significantly associated with mortality. None of these were however statistically significant on multivariate regression analysis. Adherence with anti-retroviral drugs serves to maximally suppress the virus, and when not achieved, could lead to rapid progression of HIV/AIDS, development of complications and death. Poor adherence with cART was associated with a thirteen fold increased risk of mortality in this study. Cotrimoxazole prophylaxis is known to protect against OIs including Pnemocystis jirovecii pneumonia, toxoplasmosis, bacterial pneumonia, malaria and diarrheal diseases [34]. Patients not on this are therefore prone to these infections and increased mortality. Anaemia was found to be significantly associated with mortality, in keeping with findings from other studies [10]. Anaemia can result from various causes in PLHIV, including malnutrition, OIs and drugs among others [35]. Reduced tissue oxygen delivery, hypoxia, and increased metabolic demands on the heart can predispose to mortality from anaemia.

The study has some limitations. One limitation is that it was a single hospital-based study, with the possibility of selection bias limiting the generalizability of findings. Though sepsis was considered as a distinct diagnostic entity from tuberculosis similar to other studies [10,36], there is the possibility of a bias margin in their operational definitions as some cases of tuberculosis could have presented as sepsis. However, non-tuberculous sepsis and tuberculosis when taken together, were the commonest cause of morbidity and mortality in our patients living with HIV.

Many of the patients that were involved in this study were also indigent, which limited their ability to access optimal care that might have impacted negatively on their outcome. The diagnosis of some AIDS-defining illnesses such as primary central nervous system lymphoma and pneumocystis jirovecii pneumonia were difficult to make either due to a lack of diagnostic facilities, or patients being unable to pay for them. The non-uniformity of investigations also impacted on confirming the aetiological diagnosis of sepsis. Though immunosuppression in the setting of HIV infection can predispose PLHIV to opportunistic infections and sepsis, there is the possibility that some indigent critically ill patients from other causes could fit the diagnosis of sepsis in the absence of sepsis workup and other investigations to explore differential diagnoses.

There is a need for care providers to intensify TB case findings and also preventive therapy in-order to reduce the TB burden. Attention also needs to be placed on the prevention, early diagnosis and treatment of sepsis among PLHIV. More effort should be put towards ensuring universal coverage of cART in HIV programs, with adequate counselling of clients on the importance of adherence to the medications.

## Conclusion

Findings from this study has shown that opportunistic infections and sepsis remain among the most common causes of morbidity and mortality among adult PLHIV in Nigeria despite the upscaling of cART and the changing burden of diseases in less developed countries. More attention should therefore be placed on early diagnosis, prevention of immunosuppression and sepsis through timely administration and adherence to cART and other prophylactic measures.

## Supporting information

S1 Checklist STROBE statement—Checklist of items that should be included in reports of observational studies. (DOCX)
